# Development and Evaluation of an Anti-Biotin Interference Method in Biotin-Streptavidin Immunoassays

**DOI:** 10.3390/diagnostics12071729

**Published:** 2022-07-16

**Authors:** Dong Liu, Yacob Berhane Gebreab, Jian Hu, Lili Zhou, Ning Zhang, Hui Tong, Bin Chen, Xiaoqin Wang

**Affiliations:** 1 Department of Clinical Laboratory, The First Affiliated Hospital of Xi’an Jiaotong University, Xi’an 710061, China; duguke@126.com (D.L.); yacob_b@yahoo.com (Y.B.G.); hobbyhujian@sina.com (J.H.); zhangningxjtu@163.com (N.Z.); 2 Department of Clinical Laboratory of People’s Hospital of Tongchuan, Tongchuan 727031, China; zhoulili199409@163.com (L.Z.); th290183446@163.com (H.T.); 15709101821@163.com (B.C.)

**Keywords:** biotin interference, biotin-streptavidin system, chemiluminescence assay, performance evaluation, streptavidin magnetic particles

## Abstract

The strong non-covalent interaction between biotin and streptavidin places streptavidin-based assays, used by many laboratories, at an increased risk of interference by biotin. At present, a few manufacturers have developed fully automated anti-biotin interference methods, although compared with many detection platforms, these remain insufficient. Additionally, there is a need for more methods that can achieve fully automated anti-biotin interference. We sought to develop and evaluate a new biotin interference-resisting method based on a biotin-streptavidin chemiluminescence immunoassay. Streptavidin-coated magnetic microparticles (M) of different concentrations were prepared and tested for their biotin-resistance capabilities in an automated setting (Cobas e 601). The precision, accuracy, and detection capability were also assessed. Higher concentrations of M were found to have a stronger ability to resist biotin interference. A 2.16 mg/mL concentration of M was able to resist 500 ng/mL of biotin in samples while simultaneously having a relatively weak shielding effect on the optical signals. Moreover, the total precision and accuracy of this method, designated as M3, met acceptable standards. M3 has an improved ability to resist biotin interference, can achieve full automation, and its detection performance can meet the general laboratory quality requirements.

## 1. Introduction

Chemiluminescence immunoassay (CLIA) based on biotin-avidin/streptavidin (BAS) technology has a wide range of applications with high sensitivity and a wide linear range. Approximately 85% of CLIA instruments use BAS-based analysis methods and are used by more than two-thirds of the laboratories in China [1,2,3]. The strong non-covalent interaction between biotin and streptavidin makes it desirable as a basis for many in vitro diagnostic tests. Unfortunately, this method is susceptible to interference from high biotin concentrations in blood samples [4,5,6,7]. The (exogenous) biotin in the blood sample and biotin in the reagent compete for binding sites on streptavidin reagents, and as a result, the luminescent substances cannot be captured by the streptavidin on magnetic beads, ultimately leading to a decrease in the intensity of the optical signal [8,9]. In short, biotin interference creates insufficiency of streptavidin-coated magnetic beads.

There are mainly two types of CLIA, namely sandwich and competitive CLIA, and biotin has different interference effects on these two modes. Biotin interference in a sandwich immunoassay, in which the concentration of the analyte is directly proportional to the intensity of the light signal, results in a falsely decreased result. On the other hand, the concentration of the analyte and intensity of the light signal are inversely related in the competitive immunoassay, which in turn results in a false increase in the test results. The ultimate effects of biotin interference in biotin–streptavidin-based assays are false high or false low results, which places patients at risk of unnecessary expenses and potentially harmful outcomes.

There have been several studies that sought to detect and counter biotin interference using different approaches, such as the detection of biotin concentrations in serum samples [2]; inquisition of patients regarding consumption of biotin supplements [10]; manual incubation of the serum sample with streptomycin-coated magnetic particles outside the instrument, following which the incubated supernatant is separated and used as a sample for detection [11,12]; sample dilution [13]; and reagent improvement by adding biotin antibody to reagent [14,15]. Biotin concentration detection using these methods is limited because techniques such as enzyme-linked immunosorbent assays (ELISA) and high-performance liquid chromatography (HPLC), which require additional reagents or equipment, are not widely available; investigating patients’ biotin supplementation history is time-consuming and laborious, and the reliability is poor; and it takes a long time to adsorb and neutralize biotin through streptavidin magnetic particles outside the instrument before detection, and this process is complicated and liable to large human error. Thus, the ideal anti-biotin interference method is one that has a fully automatic operation, wherein the analyte of serum sample can be directly put into the instrument for detection without any pre-treatment. In 2019, Roche [14] and Siemens [15] invented a fully automated method of anti-biotin interference through reagent improvement on the existing detection platform. However, many other detection platforms have no corresponding anti-biotin interference measures. Therefore, there may be a need for more anti-biotin interference methods for fully automated detection platforms.

Streptavidin can combine with solid phase materials without affecting its binding ability with biotin. Therefore, in BAS-based analysis, streptavidin is usually pre-combined with magnetic microparticles and then combined with biotin to facilitate their capture and separation.

The essence of biotin interference is an insufficiency of streptavidin. In this study, where “M” will be used to denoted the full term of “streptavidin-coated magnetic microparticles”, we showed that using excessive M to neutralize biotin in a sample has a useful effect in vitro. We then set out to neutralize biotin by using high concentrations of M in an automated system. Biotin has different interference effects on sandwich and competitive CLIA. The β-human chorionic gonadotropin (β-hCG) level is often detected by sandwich CLIA while the progesterone level is often detected by competitive CLIA. As number of clinical samples was large, it was easy to obtain serum samples with various concentrations of both these hormones. Therefore, β-hCG and progesterone were chosen as representatives of the sandwich and competitive methods, and the interference of biotin on detection of these two hormones using the respective CLIA method was evaluated.

## 2. Materials and Methods

### 2.1. Preparation of Experimental Materials

#### 2.1.1. Preparation of Biotin Solution

First, 10 mg of biotin lyophilized powder (purity 99.9%, catalog number B4501-1000 MG, lot number SLBS8478, Sigma-Aldrich Chemie GmbH, Schnelldorf, Germany) were dissolved in a 50 mL phosphate-buffered solution (PBS) solution (Shanghai Ruji Biotech Development Co., Ltd., Shanghai, China; Lot No.: 1906ZPW02). Using the PBS solution, we prepared biotin solutions with the following concentrations: 0, 50, 100, 200, 300, 400, 500, 600, 700, 800, 900, 1000, 2000, 3000, 4000, 5000, 10,000, and 200,000 ng/mL. Once prepared, they were stored at 4–8 °C for later use.

#### 2.1.2. Sample Preparation

In this study, we used patients’ samples that would otherwise have been discarded. These samples, with high, medium, and low concentrations of β-hCG/progesterone (Prog), were used as single or pooled serum samples. All serum samples were spiked with a 10% biotin solution. External Quality Assessment (EQA) samples were obtained from the second EQA from the Chinese National Center for Clinical Laboratories in 2019.

#### 2.1.3. Preparation of M

M (0.72 mg/mL) from Roche (Basel, Switzerland) was used as a raw material. The following concentrations of M were prepared by centrifugation (1000× *g*) for 15 min and subsequently diluted with the supernatant (0.036, 0.72, 1.44, 2.16, 2.88, 3.60, and 7.2 mg/mL, labeled from M0 to M6, respectively). These dilutions were stored at 4–8 °C in a refrigerator for later use.

#### 2.1.4. Preparation of Mixed Reagents

In this study, we mixed different types of Roche reagents (Table S1) to obtain the desired “mixture of reagents”. Mixing these different reagents would not influence our experiment in any way as the fundamental principle of detection used in this study is based on antigen–antibody reactions and, theoretically, the mixed reagents would not affect these reactions. Importantly, we were interested in observing the relationship between luminescent signals and biotin. Since CLIA is based on a sandwich method or a competitive method, we prepared two types of mixed reagents for these two methods. However, as there are more types of tests that routinely use the sandwich method, rather than the competitive method, we used 10 different types of reagents to prepare our “sandwich method mixtures R1 and R2” and five reagents to prepare “competitive method mixtures R1 and R2”. For the sandwich method, we took 2 mL of R1 of each reagent based on the Roche reagent guideline, mixed them, and put them in a sterile container marked “mixed R1 sandwich method”. Next, we took 2 mL of R2, mixed it evenly, and placed this in a separate container marked as “mixed R2 sandwich method”. Similarly, for the competitive method, we took 2.4 mL of the R1 and R2 reagents to prepare “mixed R1 competitive method” and “mixed R2 competitive method”, respectively. The reagents were stored at 4–8 °C in a refrigerator.

#### 2.1.5. Preparation of Luminescent Substances

We added 360 μL β-hCG R1 in a test tube, along with 400 μL R2 and 50 μL (β-hCG) in fresh serum (5538 IU/L). This solution mixture was incubated at 37 °C for 9 min. Next, we added 260 μL M0 (0.036 mg/mL), incubated the samples again at 37 °C for 9 min, and centrifuged this at 1000 rpm for 15 min. Thereafter, we carefully removed 700 μL supernatant and discarded it. The precipitate was used as the luminescent substance and was stored at 4–8 °C refrigerator for later use.

### 2.2. Experimental Method

In this study, a Cobas e 601 analyzer (Hitachi High-Technologies Corporation, Tokyo, Japan) was used for detection. This instrument is an Electrochemiluminescence immunoassay based on BAS, and its luminescent principle is different from CLIA. However, the principle based on BAS technology is identical. The sandwich and competitive methods used β-hCG and progesterone measurement procedures, respectively. The light transmittance was detected by a 721G spectrophotometer (Shanghai YITIAN Precision Instrument CO, LTD, Shanghai, China). Biotin concentration was detected by the Biotin ELISA Kit (catalog number EU2591, lot number 20200301, Wuhan Fine Biotech Co., Ltd., Wuhan, China) on the RT-6100 enzyme-labeling instrument (Rayto Life and Analytical Sciences Co., Ltd., Shenzhen, China).

#### 2.2.1. Evaluation of the Anti-Biotin Interference Effect of M

To assess the anti-biotin interference ability in the sandwich method, we used high-value mixed sera, and for the competitive method, we used low-value mixed sera. These mixed sera, together with 10% biotin solution at concentrations of 0 to 200,000 ng/mL, were labeled from H0 to H17 for the sandwich method and L0 to L17 for the competitive method and were used as the samples in our experiment. The previously prepared M reagents (labeled from M1 to M6) were used as reagents and used for tests with the Cobas e 601 (Roche Diagnostics). The level of and change in optical signal were observed, and a change in the amplitude of the optical signal of ±10% was considered to indicate an improved ability to resist biotin interference.

To assess the impact of the concentration of M on the optical signal level, R1 and R2 reagents were replaced with Roche’s special diluent, a luminescent substance was used as a sample, and M1–M6 were used as M reagents. The Cobas e 601 (Roche Diagnostics) was used to observe the change in light signal. 

We also assessed the light transmittance of M1–M6 at a 620-nm wavelength using a 721G spectrophotometer (Shanghai YITIAN Precision Instrument Co. Ltd., Shanghai, China) and adjusted the light transmittance to 100% with the Roche diluent.

#### 2.2.2. Evaluation of Performance

To evaluate performance, we conducted experiments with the original reagents (except for the M reagent). A 10% volume of 0, 5000, and 10,000 ng/mL biotin solutions were added to the samples. Based on the results obtained, M3 (2.16 mg/mL) was selected as the M reagent for further analyses.

Eight samples of β-hCG and progesterone (β-hCG 0.101–9432 IU/L; progesterone 0.580–171.8 nmol/L) were used to establish standard curves by means of a four-parameter logistic fit.

Precision was evaluated in accordance with the National Committee for Clinical Laboratory Standards (NCCLS) EP05-A3 document [16]. Fresh high-value and low-value sera spiked with biotin solution were used as samples, and M3 at a concentration of 2.16 mg/mL was used as the reagent. Samples were tested with the Cobas e 601 (Roche Diagnostics). A daily precision ≤1/3 of the allowable total error (TEa) (±8.33%) was considered acceptable.

To evaluate accuracy, we first performed a biotin sample recovery test based on the NCCLS EP15-A3 document [17]. After adding different concentrations of biotin solutions to high-value/low-value samples, the analyte concentrations were detected and the recovery rates calculated. Recovery rate = (measured value/expected value) × 100. The test result of the sample without biotin at M1 was set as the expected value, and other results were measured values. Recovery rates between 90% and 110% were considered as acceptable.

To assess the bias of the EQA samples [18], EQA samples with added biotin solution (samples), and M3 (M reagent) were tested in the Cobas e 601 (Roche Diagnostics). The relative bias (%) between the detected and target values were calculated. Taking relative bias (%) ≤1/2 TEa (±12.25%) as the standard, if four of five samples met the criteria, it was considered acceptable. 

We evaluated the detection ability as set out in the NCCLS EP17-A documents [19]. This comprised limit of blank (LoB), limit of detection (LoD), and limit of quantitation (LoQ).

To evaluate the LoB, we used Roche’s special diluent, with biotin solution as a blank sample. We tested these six times per day for 5 consecutive days. We selected a parameter/non-parameter test according to the data distribution, and a cutoff value containing 95% of the detection results was considered as the LoB.

Five samples, whose detection results were one to four times more than the LoB, were selected and mixed with biotin solution as samples and tested. We divided each sample into five parts, tested 1 part 5 times every day for 5 consecutive days, and obtained 25 results. According to the data distribution, using parametric/non-parametric tests, the corresponding analyte concentration was taken as the LoD when the 95% confidence limit just exceeded the LoB.

To evaluate the LoQ, a concentration corresponding to TEa ≤20% was taken as the LoQ.

To assess the anti-biotin interference ability of M3, we selected the high-, middle-, and low-value fresh samples of β-hCG and progesterone and tested the levels of biotin in the samples. Then, we detected the concentrations of β-hCG and progesterone in the original reagent (M1) and M3 modes, respectively. After adding a 10% volume of 5000 ng/mL and 10,000 ng/mL of biotin solution into the samples, the concentration of biotin in the samples was measured again, and then the concentrations of β-hCG and progesterone were measured in the M1 and M3 modes again. The concentrations of β-hCG and progesterone of M1 and M3 modes before and after biotin addition were compared.

### 2.3. Statistical Methods

GraphPad Prim 8 software (San Diego, CA, USA) was used for statistical analysis and graphing. The sample with biotin solution was calibrated with a correction coefficient of 1.1. Parametric tests were performed for data with a normal distribution, and the mean and standard deviation were calculated. Non-parametric tests were performed for non-normally distributed data, and the 95th percentile was calculated. *p* < 0.05 was considered statistically significant.

## 3. Results

### 3.1. Ability of Different Concentrations of M to Resist Biotin Interference

**Figure 1:**
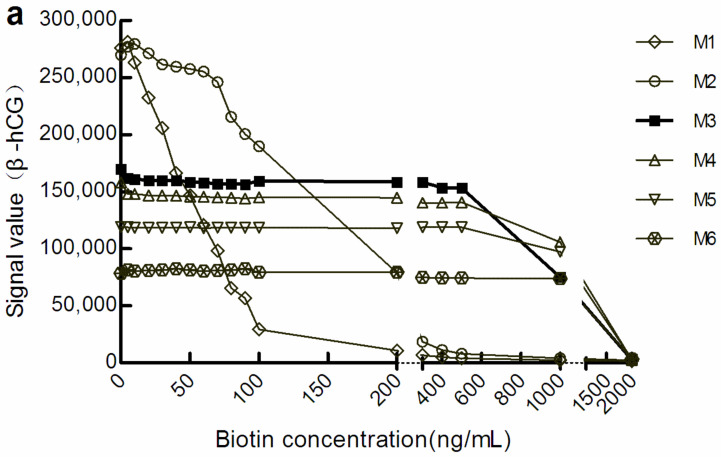

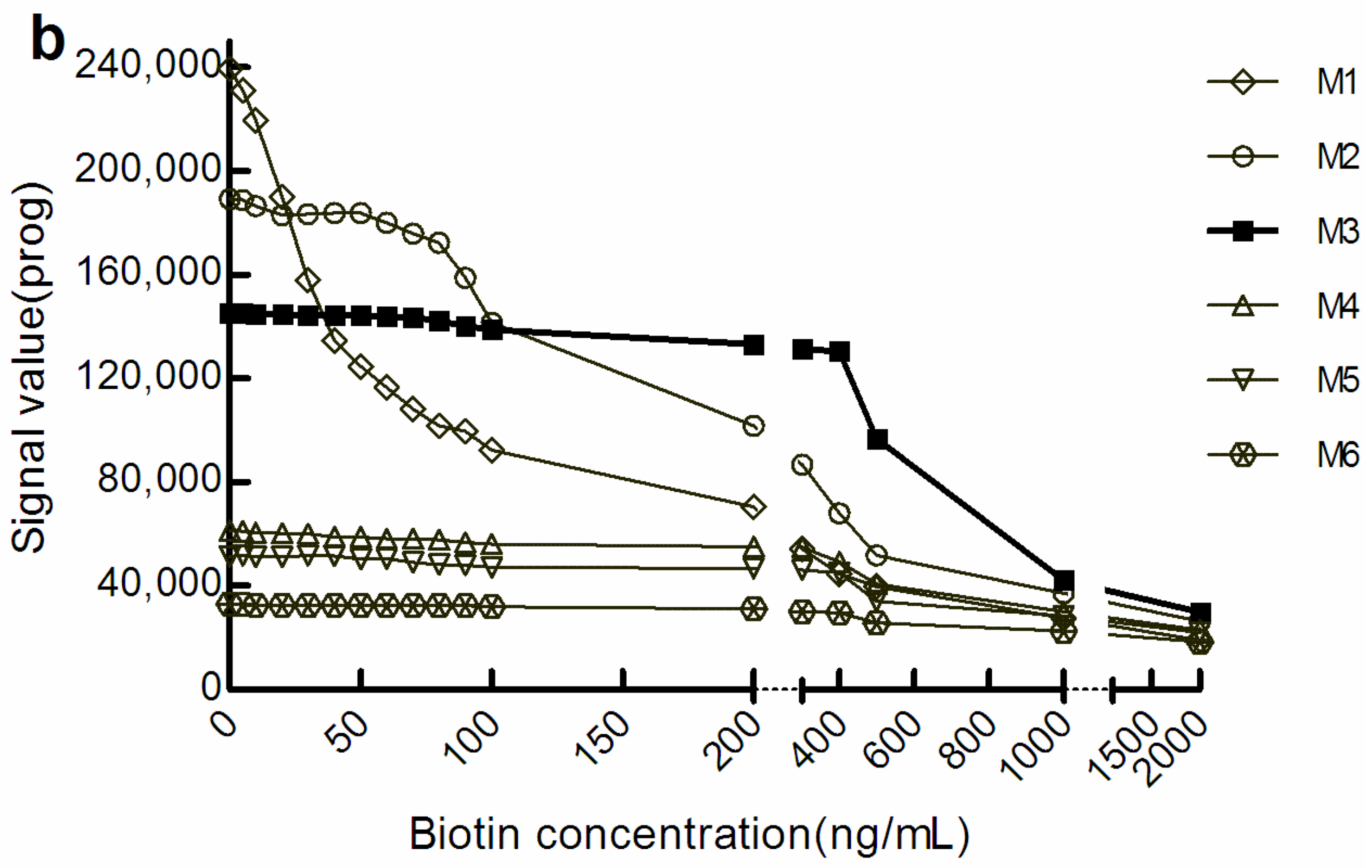
The optical signal changes with different concentrations of biotin samples. (**a**) light signal intensity in the sandwich assay; (**b**) light signal intensity in the competitive assay. The concentrations of M1 to M6 were 0.72, 1.44, 2.16, 2.88, 3.60, and 7.20 mg/mL, respectively. β-hCG: β-human chorionic gonadotropin; Prog: Progesterone.

With the increase in M concentration, the light signal level gradually decreased. However, when the light signal tended to be stable, the biotin concentration range was increased in the corresponding sample. We defined a change in the amplitude of the optical signal value within ± 10% to be good anti-biotin interference ability. According to this standard, in the sandwich mode, the biotin concentrations that M1, M3, and M6 could resist were below 20 ng/mL, below 500 ng/mL, and below 1000 ng/mL, respectively (Figure 1a). There were similar manifestations in the competition law model, although the biotin concentration range differed somewhat (Figure 1b). However, the level of optical signal decreased with the increase in the concentration of M, which suggests that increasing M concentration may increase the shielding effect on the optical signal. 

### 3.2. Shielding Effect of M on Optical Signals

#### 3.2.1. Light Signal Levels of Luminescent Substances at Different Concentrations of M

**Figure 2:**
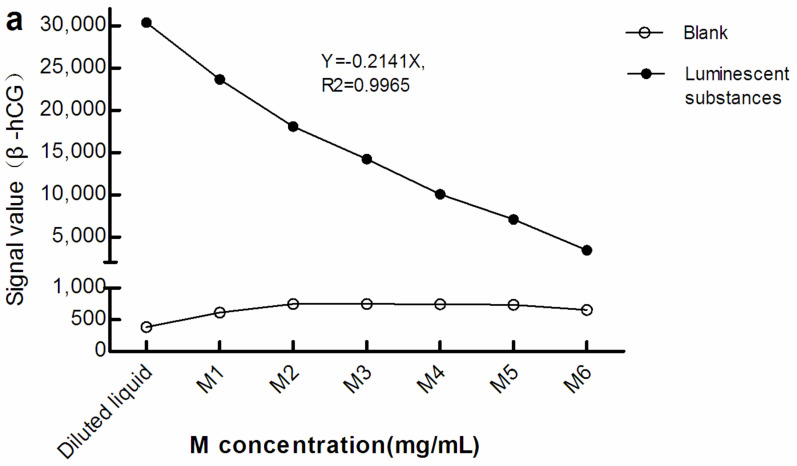

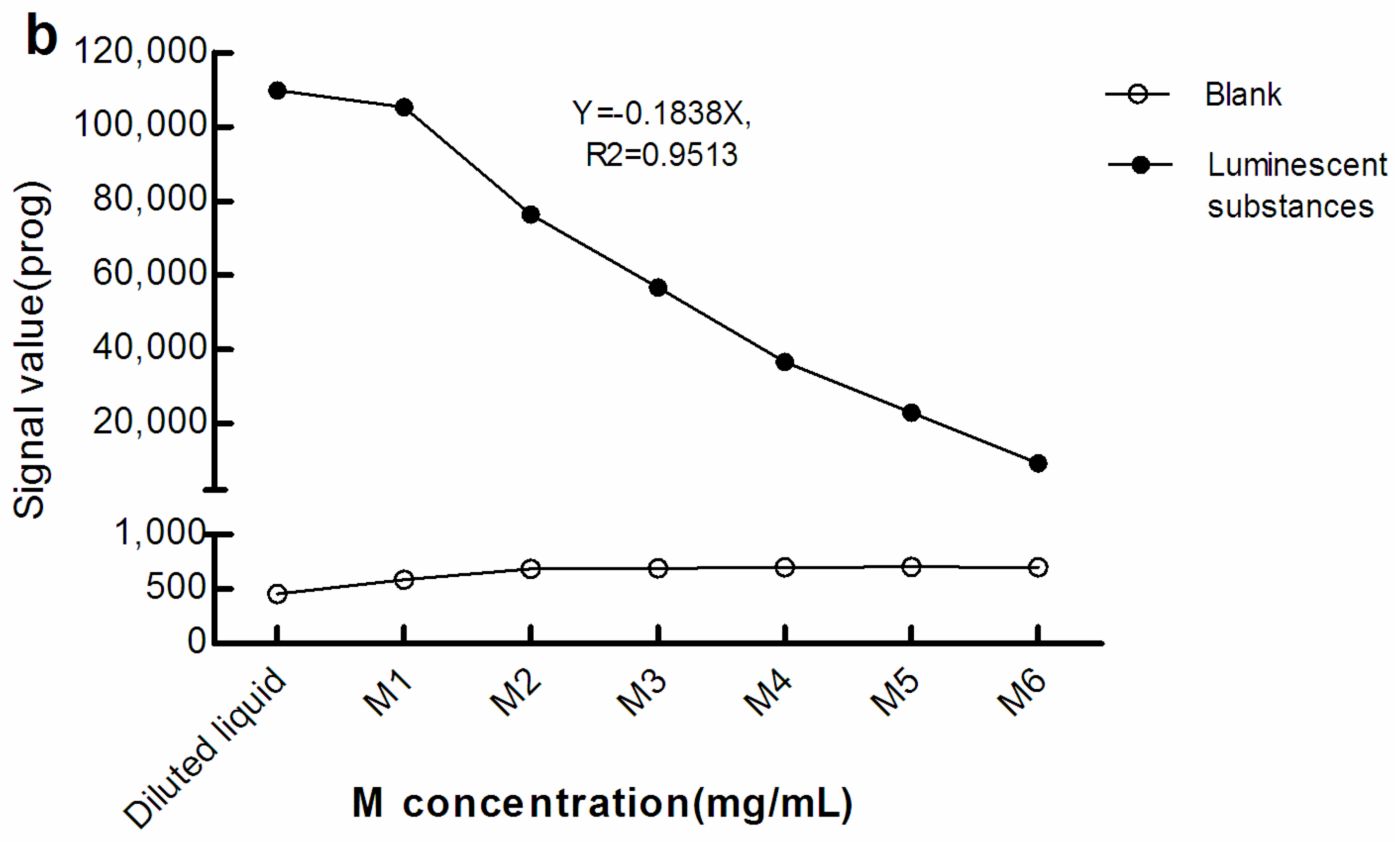
Light signal levels of luminescent materials at different concentrations of M. (**a**) sandwich method; (**b**) competitive method. The reaction system of the blank optical signal used Roche diluent as R1, R2, and the sample, and M1–M6 as M reagent. The reaction system of the light signal level of the luminescent substances was the same as the blank reaction system, except that the sample was replaced with luminescent substances. The concentration of M in the diluted liquid was 0 mg/mL. The concentrations of M1–M6 were 0.72, 1.44, 2.16, 2.88, 3.60, and 7.20 mg/mL, respectively. β-hCG: β-human chorionic gonadotropin; Prog: Progesterone.

As seen in Figure 2, with greater M concentration, the light signals in the blank (The blank reaction system used Roche diluent as R1, R2, and the sample, and M1–M6 as the M reagent. The Roche diluent was without β-hCG/Prog) increased while that of the luminescent substances diminished. Therefore, there was a negative correlation between the light signal level of the luminescent substances and concentration of M. This suggests that, when the surface area of the electrode/magnet is fixed, with increasing M concentration, too many magnetic beads may be adsorbed in a stacked state.

#### 3.2.2. Light Transmittance of M at Different Concentrations

The light transmittance was measured at 620 nm and the light transmittance decreased as the concentration of M increased (Table 1). The concentration of M correlated negatively with the logarithm based on the light transmittance value (Y = −41.45ln(X) + 93.868; R^2^ = 0.9660, *p* < 0.05). This suggests that, with increasing M concentration, the light transmittance of suspension can decrease.

### 3.3. Resistance of M3 to Biotin Interference

#### 3.3.1. Correlation between Optical and Analyte Concentration with M3

**Figure 3:**
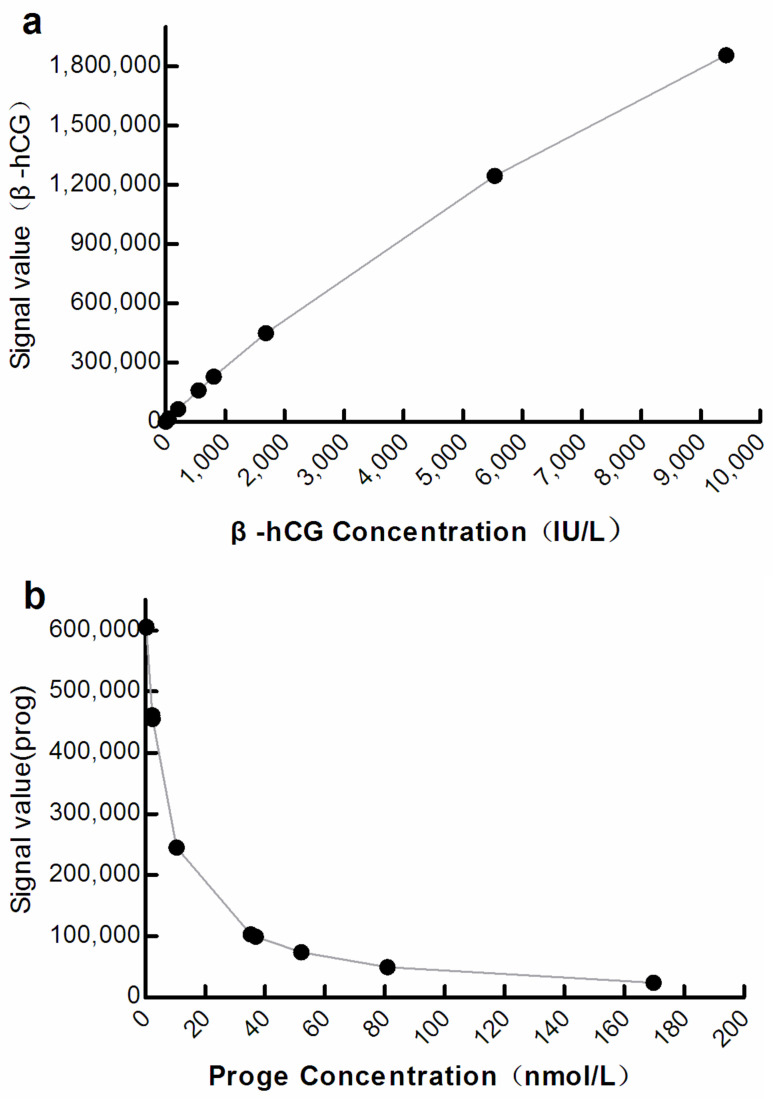
Curve showing correlation between the optical signal and analyte concentration at M3. (**a**) β-hCG: β-human chorionic gonadotropin; (**b**) Prog: Progesterone.

The optical signals correlated significantly with the analyte concentration at M3 (β-hCG: R^2^ = 0.9998, progesterone: R^2^ = 0.9838; *p* < 0.001; Figure 3). 

#### 3.3.2. Evaluation of Precision

With the increase in biotin concentration in the sample (0, 5000, and 10,000 ng/mL), although the coefficient of variation of β-hCG and Prog gradually increased, their precisions reached the acceptable standard of ≤1/3 TEa (±8.33%) (Table 2).

#### 3.3.3. Evaluation of Accuracy

The recovery rate of β-hCG and progesterone ranged between 90% and 110% when the concentration of added biotin was 5000 ng/mL. However, when the concentration of added biotin was increased to approximately 10,000 ng/mL, the recovery rate exceeded the allowable range (Table 2).

When 5000 ng/mL biotin was added, the bias of all EQA samples of β-hCG and progesterone was ≤ ½ TEa (±12.25%); however, when 10,000 ng/mL biotin was added, the bias of all samples was ≥ ½ TEa (±12.25%) (Table 3).

#### 3.3.4. Evaluation of Detection Capability

Compared with the original method (M1), the LoB, LoD, and LoQ of β-hCG and progesterone were increased in procedures using M3 (Table 4).

#### 3.3.5. Comparison of Anti-Biotin Interference Ability between M1 and M3

**Figure 4:**
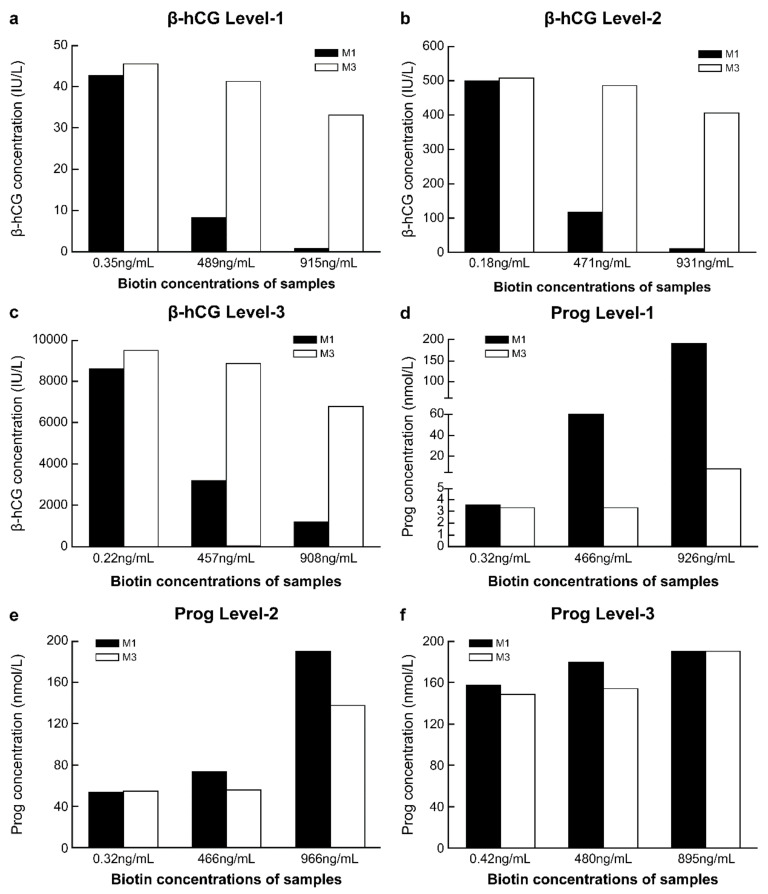
Comparison of the anti-biotin interference ability of M1 and M3. (**a**) Level-1 of β-human chorionic gonadotropin (β-hCG), with a concentration of 42.62 IU/L; (**b**) Level-2 of β-hCG, with a concentration of 499.4 IU/L; (**c**) Level-3 of β-hCG, with a concentration of 8566 IU/L; (**d**) Level-1 of progesterone (Prog), with a concentration of 3.55 nmol/L; (**e**) Level-2 of Prog, with a concentration of 54.05 nmol/L; (**f**) Level-3 of Prog, with a concentration of 157.5 nmol/L. M1, 0.72 mg/mL streptavidin-coated magnetic microparticles. M3, 2.16 mg/mL streptavidin-coated magnetic microparticles.

The concentrations of biotin in fresh serum samples were low before the addition of biotin solution, while after adding a 10% volume of biotin solution at different concentrations, the biotin concentrations in the serum samples reached approximately 500 ng/mL and 1000 ng/mL (Table S2). With the increase in the biotin concentration in the sample, compared with the detection results of M1 in samples without biotin addition, the range of variation in the detection results of β-hCG and progesterone with M1 was markedly greater than that with M3. This was true for all three biotin concentration levels. With M3, when the concentration of biotin in the sample was approximately 500 ng/mL, the detection results of β-hCG and progesterone differed by approximately 10% from those obtained with M1 in samples without biotin addition. However, with M3, when the biotin concentration in the samples was approximately 1000 ng/mL, there was more than 20% difference in the detection results of β-hCG and progesterone from the results of M1 without biotin addition (Figure 4a–e).

## 4. Discussion

Using M to resist biotin interference is a relatively simple method. At present, manual operation is required when adopting this method, which could lead to increased human error, and issues such as prolonged turnaround time [11,12,20,21]. In this study, detection was performed on the Roche platform after increasing the concentration of M, and almost no manual operation was required. This provides a reference for other detection platforms to establish convenient and fully automated anti-biotin interference methods. We showed that the higher the concentration of M, the stronger is the anti-biotin interference ability (Figure 1), while the optical signal shielding effect was also increased (Figure 1 and Figure 2, Table 1). At M3 levels, the detection performance was good (Figure 1, Figure 3 and Figure 4; Table 2 and Table 3) and met CLIA’88 (2019) [22] quality requirements.

The improved anti-biotin interference of increased M concentration in a CLIA reaction system is based on the following: the target substance is captured through the non-covalent binding of biotin to streptavidin which combines with magnetic particles. When the amount of streptavidin in the reaction system is not enough to bind all biotin in the solution, the biotin component in the reagent will compete with the biotin component in the blood for binding to streptavidin, causing interference [20]. However, when the amount of streptavidin in the reaction system exceeds the amount needed to bind to all biotins, all luminescent substances can be captured by streptavidin, and interference will not occur.

Streptavidin is usually bound to magnetic beads in detection systems. Therefore, increasing streptavidin levels implies an increase in the amount of magnetic beads. This can lead to enhanced shielding of optical signals. The diameter of magnetic beads currently commonly used in CLIA is approximately 1–5 µm (the diameter of magnetic beads used in this experiment was 2.8 μm), although the particle size of streptavidin molecules is in the nanometer range. Thus, the micron-sized magnetic beads are likely to have a greater impact on the transmission of light signals. The mechanism underlying the shielding effect may include the following: At present, in different CLIA platforms, there are two states of magnetic beads during optical signal detection. One state is that of adsorption on the electrode/magnet and the other is that of suspension. We designed two experiments to evaluate the effect of M concentration changes on optical signal values in these two states (Figure 2 and Table 1). In the first state, due to the limited surface area of the electrode/magnet, with increasing M, the surface area of the electrode/magnet was relatively insufficient, resulting in magnetic beads being adsorbed by the electrode/magnet in a stacked state; thus, the light signal of the underlying luminescent substance could not be conducted to the optical signal receiver (Figure 2). However, in the second state, due to decrease in the light transmittance of the suspension, all optical signals could not be received by optical signal receivers (Table 1). It is also possible that, due to the relative insufficient surface area of the electrode/magnet, the magnetic beads that have captured luminescent substances cannot be adsorbed and washed out. Trambas et al. [20] pointed out that the surface area of the magnet should be taken into consideration when performing an M adsorption experiment using manual methods. The shielding effect may be caused by one or more of these three reasons concurrently.

M3 has a certain anti-biotin interference ability, with a relatively small shielding effect and better detection performance (Table 2 and Table 3; Figure 3 and Figure 4). Since M3 can be self-made by residual reagents, it will not increase the economic burden on the laboratory, and its ability to be used in fully automated procedures can reduce personnel errors, without a significant increase in turn-around time. Some studies have indicated that using surplus reagents can maintain the reliability of test results [20,21]. Furthermore, the M3 program may allow manufacturers to automate the anti-biotin interference process. This is expected to improve the anti-biotin interference performance of its detection system and advance the application experience for laboratory users. For the Roche detection platform, the application of M3 will be more convenient than other platforms because, in all Roche projects, the M reagent is the same. However, in the Siemens platform, different projects have different M reagents, so it is necessary to prepare corresponding M reagents for each project separately. Therefore, we believe that using M3 ensures a simple, effective, and economical anti-biotin method at the laboratory and manufacturing scales. Thus, it may be used as a new anti-biotin interference method for the detection platform.

This study had some limitations. First, the anti-interference ability of M3 reaches only approximately 500 ng/mL, which is less than the 3510 ng/mL required by the CLSI EP37 [23]. Nevertheless, in practice, an anti-interference ability of 500 ng/mL meets most actual needs because it is very rare that the concentration of biotin in blood exceeds 100 ng/mL in outpatients [24]. A serum concentration of biotin exceeding 500 ng/mL typically only occurs with treatment using ultra-large doses of biotin, such as that used in patients with multiple sclerosis and in those with biotin-responsive basal ganglia disease [25,26,27]. At present, Roche’s anti-biotin interference method reaches only 1210 ng/mL in most of its detection items. Second, this study was performed only for β-hCG and progesterone on the Roche platform as a preliminary evaluation, and further investigations are needed for Roche’s other analytes and on other platforms. Third, this study found that magnetic beads may be the cause of optical signal shielding. Further research is necessary to determine whether the shielding effect can be reduced by reducing the particle size of the magnetic beads while retaining a sufficient level of streptavidin.

## 5. Conclusions

In conclusion, we showed that using streptavidin-bound magnetic particles at a concentration of 2.16 mg/mL (M3) improved the ability to resist biotin interference, allowed automation to a greater degree, and yielded detection performance that could meet general laboratory quality requirements. Thus, this approach could be useful as a new method of combating biotin interference.

## Figures and Tables

**Table 1:** The light transmittance at different concentrations of M.

M (mg/mL)	Transmittance (%)
M1(0.72)	100.0
M2(1.44)	85.9
M3(2.16)	66.5
M4(2.88)	54.5
M5(3.60)	35.2
M6(7.20)	8.9

M: streptavidin-coated magnetic microparticles.

**Table 2:** Precision and recovery.

Assays	Reaction Conditions/Concentration of Biotin Added to the Sample (ng/mL)	Level 1		Recovery (%)	Level 2		Recovery (%)
		Mean ± SD	CV (%)		Mean ± SD	CV (%)	
β-hCG (IU/L)	M1/0	47.06 ± 1.22	2.60	100.00	9432 ± 190.53	2.02	100.00
	M3/0	45.39 ± 0.65	1.42	96.45	9410 ± 36.56	1.90	99.77
	M3/5000	40.93 ± 1.08	2.64	86.97	8790 ± 94.45	2.31	93.19
	M3/10,000	32.61 ± 1.61	4.95	69.29	6735 ± 123.92	4.50	71.41
Prog (nmol/L)	M1/0	15.41 ± 1.00	6.47	100.00	123.8 ± 5.09	4.11	100.00
	M3/0	15.44 ± 0.29	4.26	100.19	129.9 ± 5.65	4.35	104.93
	M3/5000	15.83 ± 0.37	4.76	102.73	139.5 ± 5.17	3.71	112.68
	M3/10,000	38.85 ± 1.75	6.05	252.11	190.8 ± 0.00	0.00	>154.12

β-hCG: β-human chorionic gonadotropin, M1: 0.72 mg/mL concentration of streptavidin-coated magnetic microparticles, M3: 2.16 mg/mL concentration of streptavidin-coated magnetic microparticles, SD: standard deviation, CV: coefficient of variation, Prog: progesterone.

**Table 3:** Bias of EQA samples at different biotin concentrations.

Assays	No.	Mean ± SD	0 ng/mL Biotin Added		5000 ng/mL Biotin Added		10,000 ng/mL Biotin Added	
			Result	Bias (%)	Result	Bias (%)	Result	Bias (%)
β-hCG (IU/L)	202011	29.10 ± 2.43	28.40	−2.41	26.14	−10.17	22.12	−23.99
	202012	367.60 ± 30.63	355.10	−3.40	341.22	−7.18	287.73	−21.73
	202013	128.50 ± 10.70	123.30	−4.05	113.16	−11.94	103.55	−19.42
	202014	474.2 0± 39.50	464.50	−2.05	439.78	−7.26	369.30	−22.12
	202015	240.70 ± 20.07	233.30	−3.07	218.05	−9.41	179.58	−25.39
Prog (nmol/L)	202011	56.47 ± 4.71	57.12	1.15	62.23	10.20	168.88	299.06
	202012	22.75 ± 1.90	24.06	5.76	25.34	11.38	129.17	567.78
	202013	73.79 ± 6.12	76.19	3.25	79.12	7.22	186.03	252.11
	202014	39.92 ± 3.13	42.22	5.76	44.63	11.80	148.24	371.34
	202015	54.37 ± 4.53	56.01	3.02	59.05	8.61	165.67	304.71

β-hCG: β-human chorionic gonadotropin, EQA: External Quality Assessment, Prog: progesterone, SD: standard deviation.

**Table 4:** Detection capability of M3.

Assays	Detection Limit	M1	M3
β-HCG (IU/L)	LoB	<0.100	<0.100
	LoD	0.100	0.225
	LoQ	0.607	0.830
Prog (nmol/L)	LoB	<0.159	0.181
	LoD	0.159	0.196
	LoQ	0.636	0.831

β-hCG: β-human chorionic gonadotropin, M1: 0.72 mg/mL concentration of streptavidin-coated magnetic microparticles, M3: 2.16 mg/mL concentration of streptavidin-coated magnetic microparticles, Prog: progesterone, LoB: limit of blank, LoD: limit of detection, LoQ: limit of quantitation.

## Data Availability

The datasets generated during and/or analyzed during the current study are available from the corresponding author on reasonable request.

## Supplementary Materials

The following supporting information can be downloaded at: https://www.mdpi.com/article/10.3390/diagnostics12071729/s1, Table S1: Original reagent information and dosage for preparing mixed reagents; Table S2: Results of biotin concentration detection.
