# Supplementary material for: Development and Evaluation of an Anti-Biotin Interference Method in Biotin-Streptavidin Immunoassays
Source: Diagnostics (Basel). 2022 Jul 16;12(7):1729. doi: 10.3390/diagnostics12071729 (PMC9324022; doi:10.3390/diagnostics12071729)
Supplement: Supplementary file 1 [file diagnostics-12-01729-s001.zip › diagnostics-1744291-supplementary/Table_S1.pdf]

Table S1. Original reagent information and dosage for preparing mixed reagents.

Parameter	R1 Volume (mL)	R2 Volume (mL)	Reference No.	Lot No.
Composition of sandwich method mixed reagent (total volume: mixed R1 = 20ml, mixed R2 = 20ml)				
β-hCG	2	2	03271749190	45406003
AFP	2	2	04481798190	47350303
CA125	2	2	11776223190	48833701
CA199	2	2	11776193122	50267101
CEA	2	2	11731629322	50005801
Cyfra21-1	2	2	11820966122	45526402
NSE	2	2	12133113122	47064401
FPSA	2	2	03289788190	49578901
TPSA	2	2	08791686190	50654701
TSH	2	2	08429324190	48422903
Composition of competitive method mixed reagent (total volume: mixed R1 = 12 ml, mixed R2 = 12 ml)				
FT3	2.4	2.4	06437206190	47337201
FT4	2.4	2.4	07976836190	47808501
Prog	2.4	2.4	07092539190	47811003
T3	2.4	2.4	11731360122	48098701
T4	2.4	2.4	12017709122	47223602

β-hCG: β-human chorionic gonadotropin, AFP: Alpha 1-fetoprotein, CA125: Cancer antigen 125, CA199: Carbohydrate antigen 19-9, CEA: Carcinoembryonic antigen, FPSA: Free prostate-specific antigen, TPSA: Total prostate-specific antigen, NSE: Neuron-specific enolase, TSH: Thyroid-stimulating hormone, FT3: Free triiodothyronine, FT4: Free thyroxine, PROG: Progesterone, T3: Triiodothyronine, T4: Thyroxine.
