# Supplementary material for: Development and Evaluation of an Anti-Biotin Interference Method in Biotin-Streptavidin Immunoassays
Source: Diagnostics (Basel). 2022 Jul 16;12(7):1729. doi: 10.3390/diagnostics12071729 (PMC9324022; doi:10.3390/diagnostics12071729)
Supplement: Supplementary file 1 [file diagnostics-12-01729-s001.zip › diagnostics-1744291-supplementary/Table_S2.pdf]

Table S2. Results of biotin concentration detection

Assays	Levels	Biotin concentration (ng/mL)		
		0 ng/mL biotin added	5,000 ng/mL biotin added	10,000 ng/mL biotin added
β-hCG (IU/L)	Level-1	0.35	489	915
	Level-2	0.18	471	931
	Level-3	0.22	457	908
Prog (nmol/L)	Level-1	0.32	466	926
	Level-2	0.32	466	966
	Level-3	0.42	480	895

The concentration of β-hCG levels 1–3 are 42.62, 499.4, and 8,566 IU/L. The concentration of Prog levels 1–3 are 3.55, 54.05, and 157.5 nmol/L.
